# Submucosal Colonic Endometriosis Presenting as a Sigmoid Polyp

**DOI:** 10.14309/crj.0000000000002073

**Published:** 2026-04-09

**Authors:** Hatem Ahmed, Sameh Gomaa, Essel Dulaimi, Annapurna Korimilli

**Affiliations:** 1 Internal Medicine, Phoenixville Hospital, Phoenixville, PA; 2 Anatomic Pathology & Clinical Pathology, Phoenixville Hospital, Phoenixville, PA; 3 Gastroenterology, Penn Medicine Gastroenterology Limerick, Limerick, PA

## CASE REPORT

**Figure 1.**
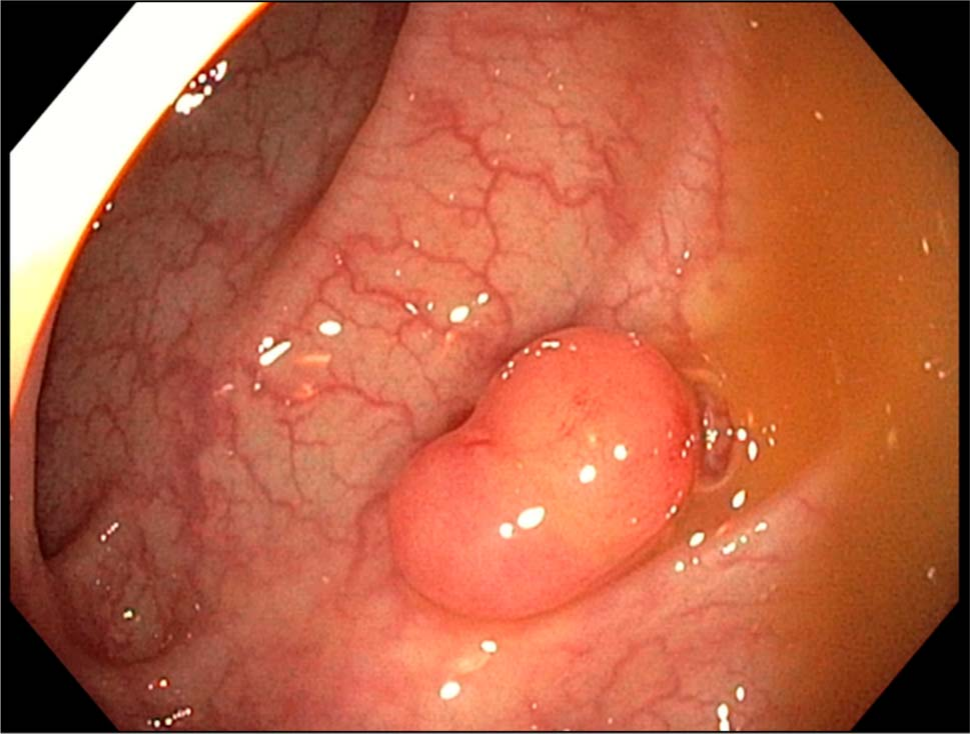
Colonoscopy revealing a solitary 18-mm sessile polyp located 30 cm from the anal verge.

**Figure 2.**
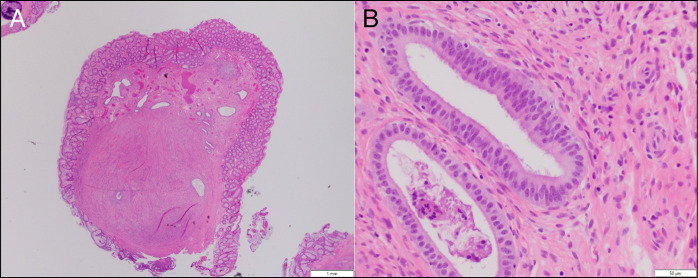
(A) Polypectomy specimen showing a submucosal nodule of endometrial-type glands and stroma elevating the colonic mucosa (H&E, original magnification 20×). (B) High-power view demonstrating endometrial-type glands and stroma within the colonic submucosa, alongside a rare mucosal endometrial gland (H&E, original magnification 400×). H&E, hematoxylin and eosin.

**Figure 3.**
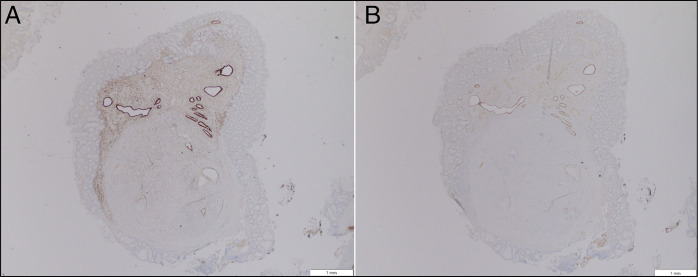
Immunohistochemistry confirming Müllerian lineage via (A) estrogen receptor strong nuclear labeling in submucosal glandular epithelium with focal stromal positivity and (B) PAX8 nuclear positivity in the submucosal glands.

**Figure 4.**
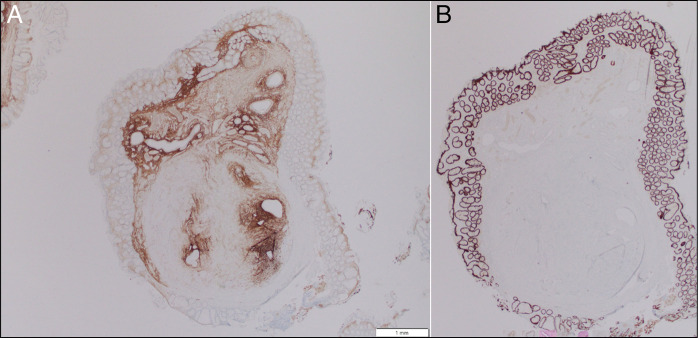
Immunohistochemistry demonstrating (A) CD10 positivity highlighting the endometrial-type stroma, and (B) CDX2 and CK20 positivity limited to native colonic mucosa with complete absence in the submucosal nodule, ruling out an intestinal origin.

Bowel endometriosis usually involves the serosa or muscularis propria; mucosal/submucosal disease presenting as a colorectal polyp is rare and is the subset most likely to be mistaken for neoplasia or colitis.^1–4^ A 49-year-old woman with remote, total hysterectomy for fibroids and no known endometriosis underwent screening colonoscopy for intermittent abdominal pain. Colonoscopy demonstrated a solitary 18-mm sessile sigmoid polyp (30 cm from the anal verge) that was removed *en bloc* with hot snare polypectomy and prophylactic clip placement (Figure 1). Histology showed a well-circumscribed submucosal nodule of endometrial-type glands and stroma elevating the overlying mucosa with hyperplastic surface changes, rare mucosal gland involvement, and no desmoplastic response (Figure 2). Immunohistochemistry confirmed Müllerian origin with ER-positive and PAX8-positive glands and CD10-positive stroma, while intestinal markers (CDX2/CK20) were absent within the lesion and highlighted adjacent native mucosa (Figures 3 and 4). No dysplasia or malignancy was identified. Awareness of this diagnostic pitfall and use of a focused immunopanel can prevent unnecessary staging or surgical referral.^3–5^ When completely excised endoscopically and without evidence of deep infiltrative disease, conservative management with routine surveillance is appropriate.^2^

## DISCLOSURES

Author contributions: H. Ahmed: writing—original draft preparation, literature research, and final approval of the manuscript. S. Gomaa: comprehensive literature review, writing—review and editing, and final approval of the manuscript. E. Dulaimi: pathological slide review, acquisition of high-quality pathology images, writing—review and editing, and final approval of the manuscript. A. Korimilli: senior supervision, patient clinical management, writing—review and editing, and final approval of the manuscript. All authors met the ICMJE criteria for authorship, including substantial contributions to the conception and design of the work, drafting and critical revision of the manuscript, and final approval of the version submitted. Hatem Ahmed, MD is the article guarantor, taking full responsibility for the integrity of the work, including its accuracy, content, and ethical considerations.

Financial disclosure: None to report.

Previous presentation: A preliminary abstract based on this work was presented as a poster at the American College of Gastroenterology Annual Scientific Meeting; October 26, 2025; Phoenix, AZ.

Informed consent was obtained for this case report.
